# Synthesis and Contractile Activity of Substituted 1,2,3,4-Tetrahydroisoquinolines

**DOI:** 10.3390/molecules16087019

**Published:** 2011-08-16

**Authors:** Iliyan Ivanov, Stoyanka Nikolova, Dimo Aladjov, Iliyana Stefanova, Plamen Zagorchev

**Affiliations:** 1 Department of Organic Chemistry, University of Plovdiv, 24 Tzar Assen Street, 4000 Plovdiv, Bulgaria; 2 Department of Biophysics, Medical University, 15A Vasil Aprilov Street, 4000 Plovdiv, Bulgaria

**Keywords:** Grignard reagent, tetrahydroisoquinolines, contractile activity

## Abstract

A series of different 1-monosubstituted and 1,1-disubstituted 1,2,3,4-tetrahydro-isoquinolines was synthesized in high yields from different ketoamides. We have developed a convenient method for the synthesis of disubstituted derivatives by interaction of ketoamides with organomagnesium compounds, followed by cyclization in the presence of catalytic amounts of *p*-toluenesulfonic acid (PTSA). A number of substituents at the C-1 in the isoquinoline skeleton were introduced varying either carboxylic acid or organomagnesium compound. Some of the obtained 1,1-dialkyl-1,2,3,4-tetrahydro-isoquinolines possess contractile activity against guinea pig’s gastric smooth muscle preparations.

## 1. Introduction

The pharmacological activity of certain tetrahydroisoquinolines has long been established [1]. The tetrahydroisoquinoline motif is present in a variety of natural products, including cactus alkaloids (peyoruvic acid) [2], mammalian alkaloids (salsoline carboxylic acid) [3,4,5], the esteinascidine family (ET743) [6,7,8,9] and spiro-benzoisoquinoline alkaloids (parfumine) [10,11]. Within the series of 1-substituted 6,7-dihydroisoquinolines there are several active sympathomimetic amines [12,13], one of which, trimetoquinol, is a potent bronchodilator [14,15]. Ohkubo and co-workers [16] synthesized a series of 1,2,3,4-tetrahydroisoquinolines, for example MK801 (disocilpine), and evaluated them for anticonvulsant activity against intracerebro-ventriculas *N*-methyl-D-aspartate (NMDA)-induced seizures in mice [17,18,19,20]. The authors [16] found that (+)-1-methyl-1-phenyl-1,2,3,4-tetrahydroiso-quinoline hydrochloride [(+)-FR115427)] was the most effective anticonvulsant, protected CA1 hippocampal neuronal degeneration in rats and also showed anti-hypoxic activity in mice. Some isoquinoline derivatives, specially 1,1-dialkyl-1,2,3,4-tetrahydroisoquinolines, have been found to have a peripheral vasodilatory effect, a sympathetic nerve stimulating effect, an analgesic effect, or an anticonvulsant effect, and a few of them have become available clinically [21]. The biological tests indicate that 1,1-dialkyl-1,2,3,4-tetrahydroisoquinolines have potent dopamine D_2_ receptor-blocking activity and an excellent safety profile. 1,1-Disubstituted tetrahydroisoquinoline derivatives, also were found in *Aristolichia* species (Aristolochiaceae). [22]. Kubota *et al*. [23] also synthesized different *N*-acyl 1,2,3,4-tetrahydroisoquinoline derivatives and evaluated their pharmacological activity as novel specific bradycardic agents.

The variety of biological activities of substituted 1,2,3,4-tetrahydroisoquinolines prompted us to synthesized a number of their derivatives. The most appropriate method for their synthesis is the Pictet-Spengler reaction. However, this classical reaction has some disadvantages, the main one of which is the ring closure after condensation of phenethylamines with an aldehyde in the classical variant (aldehydes give good yields while ketones tend not to give products at all). However, in the last several years 1,1-disubstituted tetrahydroisoquinolines have been synthesized from starting cyclic ketones using titanium(IV)isopropoxide and acetic-formic anhydride [24]. Later, Kumpaty [25] reported a selective and direct access to secondary amines by reductive mono-*N*-alkylation of primary amines in the presence of the Ti(*i*-PrO)_4_ and NaBH_4_. A new, environmentally friendly variation of the Pictet-Spengler reaction has been elaborated using a small pore size zeolite, Ersorb 4 [26]. Some authors have reported the synthesis and an application of a new planar-chiral Lewis acid based on a 1,2-azaborolyl framework [27]. Pictet-Spengler condensation of dopamine with (+)-menthyl pyruvate followed by acid hydrolysis furnished (-)-*R*-salsolinol-1-carboxylic acid in good yield [28,29,30]. The basic method, described in literature is preparation of 1,l-dimethyltetrahydroisoquinoline from 3,4-dihydro-6,7-dimethoxy-1-methylisoquinoline [31]. Recently Kałuza and co-workers described a facile synthesis of highly substituted, optically pure tetraydroisoquinolines with a quaternary carbon stereocenter [32]. Funabashi *et al*. [33] reported the first example of a catalytic enantioselective quaternary stereocenter construction through a Reissert-type reaction with quinolines, using a bifunctional catalyst. 1-Substituted isoquinolines or 3,4-dihydroisoquinolines were used as starting materials for the synthesis of Reisert compounds [34,35,36,37,38]. Stereodivergent synthesis of 1,10-*cis*- and -*trans*-thiazolo[4,3-a]isoquinolinones, starting from *N*-4,3-dimethoxyphenethylthiazolidinedione and using *N*-acyliminium ion or Parham cyclization, also was reported recently [39]. Kirkpatrick and Maclaren prepared 1,1,-disubstituted 1,2,3,4-tetrahydro-b-carbolines by action of trifluoracetic acid on enamines of tryptamine or tryptophan [40]. Later Bobowski [41,42] reported condensation of 1*H*-indole-3-ehtanamines with different 2,4-pentanediones and b-keto esters, followed by acid-catalyzed ring closure of resulting enamines to corresponding 1,1-disubtituted indoles.

## 2. Results and Discussion

The biological activity of isoquinoline derivatives, as analogues of various drugs, has provided great deal of interest for the synthesis of new compounds. Papaverine, for example, is a smooth muscle relaxant and vasodilator which acts directly on the heart muscle. The biological activity of papaverine attracted a great deal of our interest for the synthesis and investigations of 1-substituted isoquinoline derivatives, as potential new drugs.

**Scheme 1:**
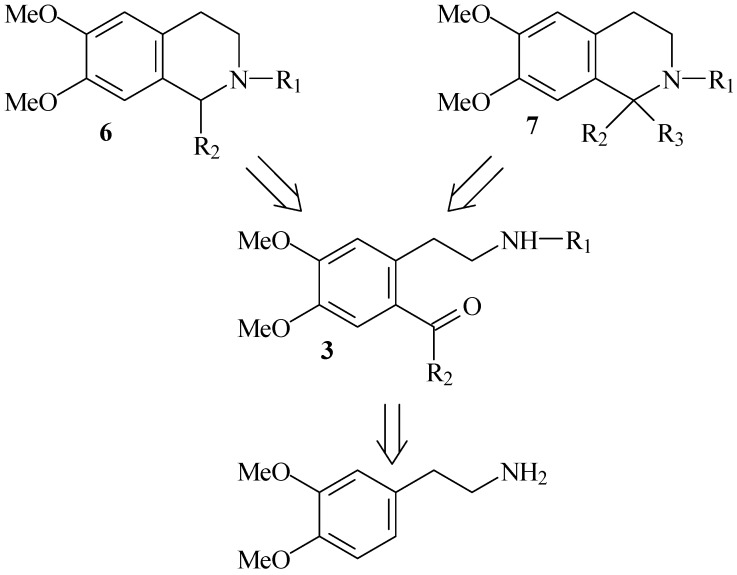
Retrosynthetic scheme for the synthesis of both 1-monosubstituted and 1,1-disubstituted 1,2,3,4-tetrahydroisoquinolines.

We report herein an alternative of the classical methods which includes *ortho*-acylation of 2-phenethylamines in polyphosphoric acid and following cyclization. In our previous reports we applied this protocol for the synthesis of variety *O*- and *N*-heterocycles and alkaloids [43,44,45,46,47]. Our synthetic approach to 1- and 1,1-disubstituted 1,2,3,4-tetrahydroisoquinolines is depicted in the Scheme 1, which shows the key steps as well as the main starting material.

**Scheme 2:**
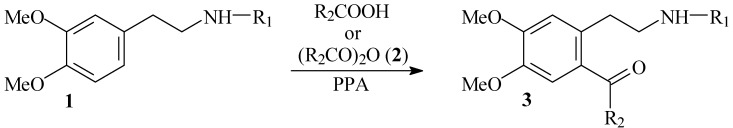
Synthesis of starting ketoamides.

Our strategy is based on the acylation of the amides of homoveratrylamine **1** with carboxylic acids and application of obtained ketoamides **3 **for the preparation of 1-substituted **6** or 1,1-disubstited 1,2,3,4-tetrahydroisoquinolines **7**. Starting ketoamides of homoveratrylamine **3** were obtained by Friedel-Crafts-type acylation. The Friedel-Crafts acylation of activated benzene rings in the presence of polyphosphoric acid (PPA) is a very convenient method for direct synthesis of aromatic ketones [43], 1-substituted 3,4-dihydroisoquinolines [44], 1-substituted 3,4-dihydro-b-carbolines [45], quinazolinones [46], isochromanes [47], *etc*. The reaction of amides of homoveratrylamine **1** with acetic anhydride, benzoic or phenylacetic acid **2 **in PPA gave the expected ketoamides **3** in high yield (Scheme 2, Table 1).

**Table 1:** Reaction conditions and yields for starting ketoamides **3**.

3	R_1_	R_2_	Reaction conditions	mp, °C	Yield [%]
**a**	СОCH_3_	CH_3_	2 h, 80 °С	124–125	92
**b**	COC_6_H_5_	CH_3_	2 h, 80 °С	147–148	80
**c**	COCH_2_C_6_H_5_	CH_3_	2 h, 80 °С	135–137	79
**d**	COOC_2_H_5_	CH_3_	3 h, 80 °С	90–90.5	95
**e**	SO_2_CH_3_	CH_3_	2 h, 80 °С	140–141	75
**f**	CONHC_6_H_5_	CH_3_	2 h, 60 °С	118–121	75
**g**	СОCH_3_	C_6_H_5_	2 h, 80 °С	212–213	87
**h**	COC_6_H_5_	C_6_H_5_	2 h, 80 °С	117–121	85
**i**	COCH_2_C_6_H_5_	C_6_H_5_	4 h, 60 °С	108–111	89
**j**	COOC_2_H_5_	C_6_H_5_	3 h, 80 °С	97–98	89
**k**	SO_2_CH_3_	C_6_H_5_	3h, 80 °C	84–86	83
**l**	CONHC_6_H_5_	C_6_H_5_	4 h, 60 °С	128–131	76
**m**	СОCH_3_	CH_2_C_6_H_5_	20 h, 60 °С	188–189	62
**n**	COC_6_H_5_	CH_2_C_6_H_5_	20 h, 60 °С	141–141.5	82

**Scheme 3:**
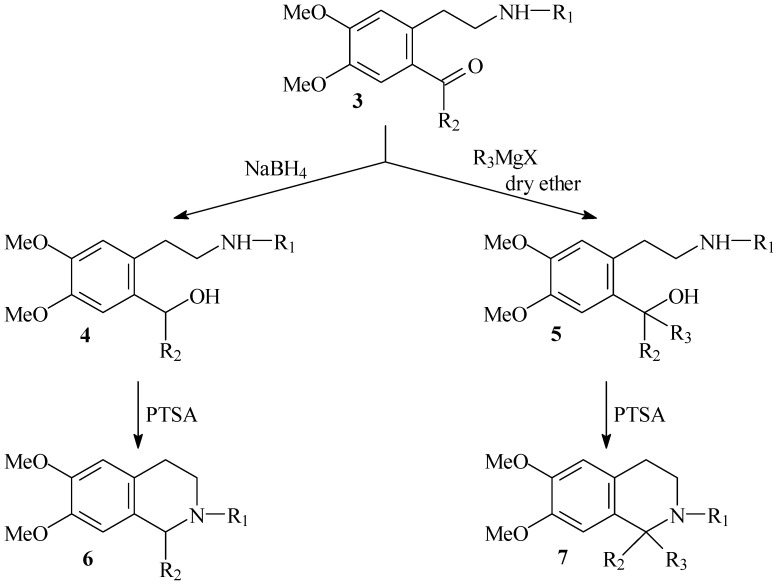
Synthesis of 1-substituted and 1,1-disubstituted-1,2,3,4-tetrahydroisoquinolines.

The next step in our synthesis was application of acylated ketoamides for the construction of isoquinoline ring system. We anticipated that 1-substituted 1,2,3,4-tetrahydroisoquinolines could be prepared trough reduction of ketoamides followed by cyclization of newly obtained hydroxyamides through *p-*toluenesulfonic acid. Respectively, 1,1-disubstituted 1,2,3,4-tetrahydroisoquinolines could be prepared from **4** and **5**, obtained from ketoamides and Grignard reagents (Scheme 3).

For the next step, **3** were reduced with NaBH_4_ in methanol to give corresponding **4 **with 85–90% yields (Table 2). 

**Table 2:** Synthesis of hydroxyamides **4**.

4	R_1_	R_2_	Yield [%]	mp, °C
**a**	СОCH_3_	CH_3_	95	108–110
**b**	COC_6_H_5_	CH_3_	94	109–110
**c**	COCH_2_C_6_H_5_	CH_3_	92	oil
**d**	COOC_2_H_5_	CH_3_	92	85–87
**e**	SO_2_CH_3_	CH_3_	90	oil
**f**	CONHC_6_H_5_	CH_3_	91	147–150
**g**	СОCH_3_	C_6_H_5_	94	133–135
**h**	COC_6_H_5_	C_6_H_5_	90	58–60
**i**	COCH_2_C_6_H_5_	C_6_H_5_	91	oil
**j**	COOC_2_H_5_	C_6_H_5_	91	92–95
**k**	SO_2_CH_3_	C_6_H_5_	90	oil
**l**	CONHC_6_H_5_	C_6_H_5_	90	73–75
**m**	СОCH_3_	CH_2_C_6_H_5_	95	oil
**n**	COC_6_H_5_	CH_2_C_6_H_5_	91	117–118

Compounds **5** were prepared with good (50–56%) yields from starting ketoamides **3** and 5-fold excess of magnesium and equimolar amounts of alkyl- (or aryl-) halide. Reaction proceeded at room temperature in dry ether (Table 3).

**Table 3:** Reaction of ketoamides with Grignard reagents.

5	R_1_	R_2_	R_3_	Yield [%]	mp, °C
**a**	СОCH_3_	CH_3_	CH_3_	52	oil
**b**	COC_6_H_5_	CH_3_	CH_3_	50	160–162
**c**	COCH_2_C_6_H_5_	CH_3_	CH_3_	50	oil
**d**	COOC_2_H_5_	CH_3_	CH_3_	- *	-
**e**	SO_2_CH_3_	CH_3_	CH_3_	- *	-
**n**	COC_6_H_5_	CH_2_C_6_H_5_	CH_3_	50	48–50
**o**	СОCH_3_	CH_3_	C_2_H_5_	- *	-
**p**	COC_6_H_5_	CH_3_	C_2_H_5_	65	131–135
**r**	SO_2_CH_3_	CH_3_	C_2_H_5_	- *	-
**s**	COC_6_H_5_	CH_3_	C_6_H_5_	50	96–98
**t**	SO_2_CH_3_	C_6_H_5_	C_2_H_5_	51	131–134

* compounds were directly cyclized.

The next step was cyclization of the newly synthesized compounds **4** and **5**. We found that **4** in the presence of a catalytic amount of toluene-*p*-sulfonic acid for 30 min at rt in dichloromethane afforded the corresponding 1,2,3,4-tetrahydroisoquinolines **6** with high yield 90–97% (Table 4).

**Table 4:** Synthesis of 1-substituted 1,2,3,4-tetrahydroisoquinolines.

6	R_1_	R_2_	Yield [%]	mp, °C
**a**	СОCH_3_	CH_3_	94	97–98
**b**	COC_6_H_5_	CH_3_	90	126–127
**c**	COCH_2_C_6_H_5_	CH_3_	92	115–116
**d**	COOC_2_H_5_	CH_3_	90	72–74
**e**	SO_2_CH_3_	CH_3_	92	105–106
**f**	CONHC_6_H_5_	CH_3_	91	178–180
**g**	СОCH_3_	C_6_H_5_	94	109–191
**h**	COC_6_H_5_	C_6_H_5_	93	143–144
**i**	COCH_2_C_6_H_5_	C_6_H_5_	95	oil
**j**	COOC_2_H_5_	C_6_H_5_	88	oil
**k**	SO_2_CH_3_	C_6_H_5_	90	183–184
**l**	CONHC_6_H_5_	C_6_H_5_	89	120–122
**m**	СОCH_3_	CH_2_C_6_H_5_	91	102–105
**n**	COC_6_H_5_	CH_2_C_6_H_5_	94	189–192

The same protocol can be readily used for the cyclization to 1,1-disubstituted 1,2,3,4-tetrahydro-isoquinolines **7** (Table 5).

**Table 5:** Cyclisation to 1,1-disubstituted 1,2,3,4-tetrahydroisoquinolines.

7	R_1_	R_2_	R_3_	Yield [%]	mp, °C
**a**	СОCH_3_	CH_3_	CH_3_	96	123–125
**b**	COC_6_H_5_	CH_3_	CH_3_	97	143–146
**c**	COCH_2_C_6_H_5_	CH_3_	CH_3_	97	oil
**d**	COOC_2_H_5_	CH_3_	CH_3_	90	55-56
**e**	SO_2_CH_3_	CH_3_	CH_3_	95	123–125
**n**	COC_6_H_5_	CH_2_C_6_H_5_	CH_3_	30	164–166
**o**	СОCH_3_	CH_3_	C_2_H_5_	60	76–81
**p**	COC_6_H_5_	CH_3_	C_2_H_5_	80	90–92
**r**	SO_2_CH_3_	CH_3_	C_2_H_5_	60	100–102
**s**	COC_6_H_5_	CH_3_	C_6_H_5_	60	103–133
**t**	SO_2_CH_3_	C_6_H_5_	C_2_H_5_	60	138–141

**Scheme 4:**
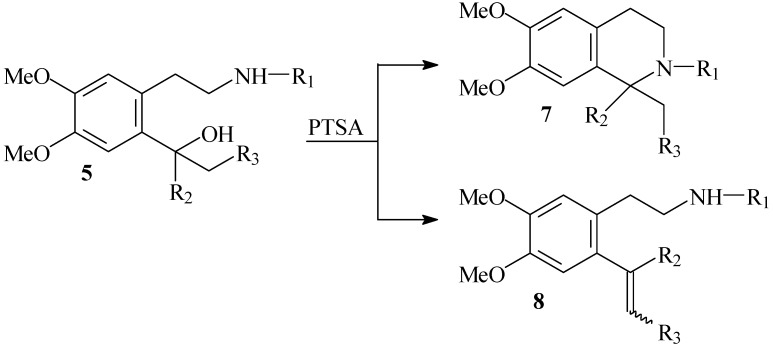
Formation of styrenes **8**.

We also found that when the substituent at the C-1 is ethyl or benzyl (in some cases also methyl), the styrene products **8** were formed also than expected cyclic 1,1-disubtituted product **7** (Scheme 4, Table 6). 

**Table 6:** Formation of styrenes.

8	R_1_	R_2_	R_3_	Yield [%]	mp, °C
**g**	COC_6_H_5_	CH_3_	CH_3_	65	72–75
**n**	COC_6_H_5_	CH_3_	C_6_H_5_	50	48–50
**s**	COC_6_H_5_	C_6_H_5_	H	50	96–98
**t**	SO_2_CH_3_	C_6_H_5_	CH_3_	30	145–148

### 2.1. Estimation of Gastric Smooth Muscle Contractile Activity for Some of the Newly Synthesized Compounds

The experiments were performed on gastric corpus smooth muscle preparations obtained from adult male guinea-pig. All experimental procedures were done in strict accordance with the current European regulations (86/609/EEC) regarding the protection of animals used for experimental purposes. The spontaneous contractile activity of the smooth-muscle strips were measured with the help of a tensotransducer measuring system at isometric conditions.

**Figure 1:**
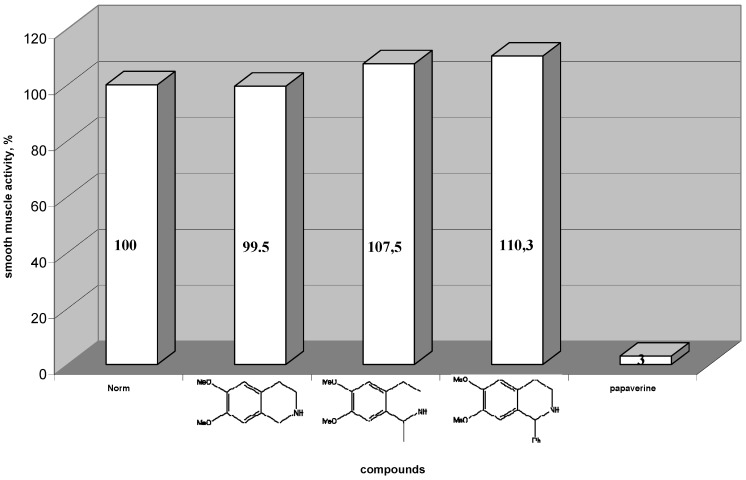
Change of spontaneous contractile activity of gastric smooth muscles preparation after using 1-substituted 1,2,3,4-tetrahydroisoquinoline derivatives and papaverine, normal activity is taken for 100%.

The biological activity of papaverine attracted a great deal of our interest for the synthesis and investigations of 1- and 1,1-disubstituted isoquinoline derivatives, as potential new drugs. The target compounds, being structural analogues of known bioactive leads, as papaverine and cryptostiline, are expected to show biological activity. For this purpose we tested three main groups of compounds for contractile activity. In search of the reason for activity, firstly we estimated 1,2,3,4-tetrahydroisoquinoline skeleton and 1-methyl- and 1-phenyl-6,7-dimetoxy-1,2,3,4-tetrahydroisoquinolines. We found that the absence of substituents devoided the compounds of contractile activity, as shown in Figure 1. The isoquinoline derivatives were less effective in contractile smooth muscle activity (−0.5%, +7.5% and +10.3% *vs.* control, respectively).

**Figure 2:**
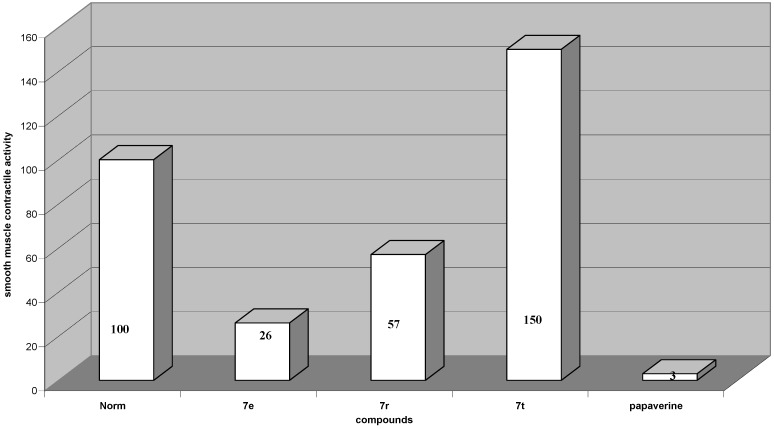
Change of spontaneous contractile activity of gastric smooth muscles preparation after using 1,1-disubstituted 1,2,3,4-tetrahydroisoquinoline derivatives and papaverine, normal activity is taken for 100%.

The second group included 1,1-disubstituted-6,7-dimetoxy-1,2,3,4-tetrahydroisoquinolines. The estimation of contractile activity showed that tested compounds have similar effect, as papaverine. The isoquinoline derivatives 7e and 7r were most effective in contractile smooth muscle activity (−74% and −43% *vs*. control, respectively) (Figure 2).

**Figure 3:**
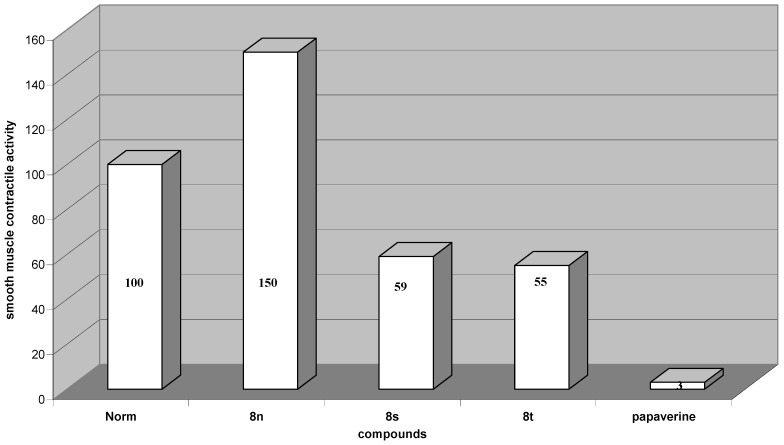
Change of spontaneous contractile activity of gastric smooth muscles preparation after using styrenes and papaverine, normal activity is taken for 100%.

The third group included styrene products **8**. As shown in Figure 3, styrenes 8s and 8t were most effective in contractile smooth muscle activity (−41% and −45% *vs.* control, respectively) (Figure 3). The contractile activity against smooth muscle preparations of these compounds were not as high as the activity of **7**.

## 3. Experimental

Reagents and chemicals were purchased from commercial sources (Sigma-Aldrich S.A. and Riedel-de Haën) and used as received. Melting points were determined on a Boetius hot stage apparatus and are uncorrected. Spectra were recorded on a Bruker Avance DRX250 spectrometer. ^1^H-NMR and ^13^C-NMR spectra were taken in CDCl_3_ (unless otherwise specified) at 250 or 600 MHz and 62.5 MHz respectively. Chemical shifts were given in part per million (ppm) relative and were referenced to TMS (δ = 0.00 ppm) as an internal standard and coupling constants are indicated in Hz. All the NMR spectra were taken at rt (ac. 295 K). Elemental analyses were performed with a TruspecMicro. TLC was carried out on precoated 0.2 mm Fluka silica gel 60 plates, using diethyl ether:n-hexane:1:1 as the eluent system. Merck silica gel 60 (0.063–0.2 mm) was used for column chromatographic separation. Polyphosphoric acid was obtained from 85% phosphoric acid and P_2_O_5_ (1:1 w/w).

### 3.1. Typical Procedure for Preparation of N-[2-(2-Acyl-4,5-dimethoxyphenyl)-ethyl]amides ***3***

To a solution of amide **1** (3 mmol) and the corresponding carboxylic acid or their anhydrides **2** (5 mmol) in dichloromethane (10 mL) in an open flask polyphosphoric acid (7 g) was added. The mixture was stirred on a mechanical stirrer carefully at 60 °C or 80 °C then poured on crushed ice and extracted with CH_2_Cl_2_ (3 × 20 mL). The combined extracts were dried (Na_2_SO_4_), filtered on the short column filled with neutral Al_2_O_3_ and then concentrated.

*N-(2-Acetyl-4,5-dimethoxyphenethyl)acetamide* (**3a**): known compound [53,54,55,56].

*N-(2-Acetyl-4,5-dimethoxyphenethyl)benzamide* (**3b**): ^1^H-NMR: 2.61 (s, 3H), 3.10 (t, *J =* 6.4, 2H), 3.75 (dt, *J =* 6.6, 5.4, 2H), 3.88 (s, 3H), 3.90 (s, 3H), 6.80 (broad s, 1H, NH), 7.16 (s, 1H), 7.28 (s, 1H), 7.34–7.47 (m, 4H), 7.77–7.80 (m, 1H); ^13^C-NMR: 201.5, 181.6, 167.4, 152.1, 146.8, 134.5, 131.0, 128.3, 126.9, 114.0, 112.6, 56.1, 55.9, 42.4, 32.1, 29.4. Anal. calcd. for C_19_H_21_NO_4_: C, 69.71; H, 6.47; N, 4.28. Found: C, 69.87; H, 6.58; N, 4.18.

*N-(2-Acetyl-4,5-dimethoxyphenethyl)-2-phenylacetamide* (**3c**): ^1^H-NMR: 2.51 (s, 3H), 2.95 (t, *J =* 6.8, 2H), 3.47 (s, 2H), 3.51 (dd, *J* = 7.0, 5.2, 2H), 3.89 (s, 3H), 3.92 (s, 3H), 6.43 (broad s, 1H, NH), 6.70 (s, 1H), 7.12 (s, 1H), 7.14–7.27 (m, 5H); ^13^C-NMR: 200.3, 181.6, 171.2, 152.0, 146.7, 135.0, 134.3, 129.3, 128.7, 128.6, 126.9, 114.1, 112.97, 56.2, 55.97, 43.8, 41.5, 32.5, 29.2. Anal. calcd. for C_20_H_23_NO_4_: C, 70.36; H, 6.79; N, 4.10. Found: C, 70.48; H, 6.95; N, 3.90.

*Ethyl 2-acetyl-4,5-dimethoxyphenethylcarbamate* (**3d**): ^1^H-NMR: 1.21 (t, *J* = 7.1, 3H), 2.58 (s, 3H), 3.03 (t, *J =* 6.9, 2H), 3.43 (dd, *J =* 12.7, 6.6, 2H), 3.92 (s, 3H), 3.93 (s, 3H), 4.08 (q, *J =* 7.1, 2H), 5.31 (broad s, 1H, NH), 6.75 (s, 1H), 7.22 (s, 1H); ^13^C-NMR: 200.0, 156.7, 151.8, 146.8, 134.4, 129.7, 114.3, 113.2, 60.5, 56.3, 55.9, 42.5, 33.9, 29.3, 14.6. Anal. calcd. for C_15_H_21_NO_5_: C, 61.00; H, 7.17; N, 4.74. Found: C, 61.25; H, 7.45; N, 4.54.

*N-(2-Acetyl-4,5-dimethoxyphenethyl)methanesulfonamide* (**3e**): ^1^H-NMR (DMSO): 2.53 (s, 3H), 2.82 (s, 3H), 2.94 (t, *J* = 7.2, 2H), 3.12 (dd, *J* = 7.4, 6.1, 2H), 3.80 (s, 3H), 3.81 (s, 3H), 6.89 (s, 1H), 6.98 (t, *J* = 5.6, 1H, NH), 7.35 (s, 1H); ^13^C-NMR: 200.6, 151.5, 146.8, 133.1, 129.7, 115.1, 114.1, 56.1, 55.9, 44.2, 39.6, 34.1, 29.8. Anal. calcd. for C_13_H_19_NO_5_S: C, 51.81; H, 6.35; N, 4.65; S, 10.64. Found: C, 51.96; H, 6.15; N, 4.72; S, 10.45.

*1-(2-Acetyl-4,5-dimethoxyphenethyl)-3-phenylurea* (**3f**): ^1^H-NMR: 2.42 (s, 3H), 2.56 (s, 1H, CONH), 2.91 (dd, *J* = 6.5, 7.9, 2H), 3.28 (td, *J* = 6.4, 7.3, 2H), 3.73 (s, 3H), 3.75 (s, 3H), 5.78 (t, *J* = 5.8, 1H, NHCO), 6.64 (s, 1H), 6.77 (tt, *J* = 1.1, 7.7, 1H), 7.05 (dd, *J* = 1.6, 6.8, 1H), 7.09 (s, 1H), 7.26 (dd, *J* = 3.4, 5.6, 2H), 7.83 (s, 1H); ^13^C-NMR: 199.7, 156.1, 151.7, 146.6, 140.0, 134.7, 129.3, 128.7, 121.7, 118.6, 114.6, 113.4, 56.1, 55.9, 41.3, 34.8, 29.4. Anal. calcd. for C_19_H_22_N_2_O_4_: C, 66.65; H, 6.48; N, 8.18. Found: C, 66.86; H, 6.28; N, 8.32.

*N-(2-Benzoyl-4,5-dimethoxyphenethyl)acetamide* (**3g**): ^1^H-NMR: 1.95 (s, 3H), 2.86-2.88 (m, 2H), 3.59–3.52 (m, 2H), 3.80 (s, 3H), 3.97 (s, 3H), 6.84 (s, 1H), 6.89 (s, 1H), 7.01 (broad s, 1H, NH), 7.54–7.45 (m, 2H), 7.66–7.58 (m, 1H), 7.85–7.81 (m, 2H); ^13^C-NMR: 198.2, 170.6, 151.4, 146.5, 138.0, 133.4, 130.5, 130.3, 129.96, 128.5, 128.3, 113.3, 112.9, 56.2, 56.1, 41.9, 31.8, 23.2. Anal. calcd. for C_19_H_21_NO_4_: C, 69.71; H, 6.47; N, 4.28. Found: C, 69.98; H, 6.29; N, 4.34.

*N-(2-Benzoyl-4,5-dimethoxyphenethyl)benzamide* (**3h**): known compound [48].

*N-(2-Benzoyl-4,5-dimethoxyphenethyl)-2-phenylacetamide* (**3i**): ^1^H-NMR: 2.84 (t, *J* = 6.7, 2H), 3.51 (s, 2H), 3.56–3.60 (m, 2H), 3.80 (s, 3H), 3.95 (s, 3H), 6.76 (broad s, 1H, NH), 6.80 (s, 1H), 6.84 (s, 1H), 7.29–7.15 (m, 5H), 7.52–7.46 (m, 2H), 7.62–7.59 (m, 1H), 7.77–7.74 (m, 2H); ^13^C-NMR: 197.5, 171.3, 151.3, 146.3, 138.1, 135.1, 133.1, 130.4, 129.9, 129.3, 128.6, 128.4, 126.9, 113.2, 113.1, 56.1, 56.0, 43.8, 41.6, 31.6. Anal. calcd. for C_25_H_25_NO_4_: C, 74.42; H, 6.25; N, 3.47. Found: C, 74.78; H, 6.37; N, 3.25.

*Ethyl 2-benzoyl-4,5-dimethoxyphenethylcarbamate* (**3j**): known compound [44].

*N-(2-Benzoyl-4,5-dimethoxyphenethyl)methanesulfonamide* (**3k**): ^1^H-NMR: 2.79 (s, 3H), 2.94 (t, *J =* 6.6, 2H), 3.48 (ddd, *J =* 2.2, 5.9, 6.5, 2H), 3.79 (s, 3H), 3.99 (s, 3H), 5.66 (t, *J* = 5.1, 1H, NH), 6.87 (s, 1H), 6.91 (s, 1H), 7.49 (tdd, *J* = 1.4, 6.6, 8.2, 2H), 7.59–7.66 (m, 1H), 7.79 (t, J=1.8, 1H), 7.82 (t, *J* = 1.4, 1H); ^13^C-NMR: 197.7, 151.5, 146.6, 138.0, 133.2, 132.6, 130.4, 130.1, 128.4, 113.5, 58.7, 56.1, 45.0, 39.7, 32.9. Anal. calcd. for C_18_H_21_NO_5_S: C, 59.49; H, 5.82; N, 3.85; S, 8.82. Found: C, 59.70; H, 5.95; N, 3.64; S, 8.61.

*1-(2-Benzoyl-4,5-dimethoxyphenethyl)-3-phenylurea* (**3l**): ^1^H-NMR: 2.87 (t, *J* = 6.9, 2H), 3.51 (q, *J* = 6.8, 2H), 3.74 (s, 3H), 3.90 (s, 3H), 5.92 (t, *J* = 4.9, 1H, NHCO), 6.83 (s, 1H), 6.87 (s, 1H), 6.99 (tt, *J* = 1.3, 7.7, 1H), 7.08 (broad s, 1H, CONH), 7.18–7.31 (m, 4H), 7.44 (tt, *J* = 1.4, 6.8, 2H), 7.54–7.61 (m, 1H), 7.74–7.77 (m, 2H); ^13^C-NMR: 198.0, 156.0, 151.4, 146.4, 139.1, 138.2, 133.6, 133.1, 130.4, 129.9, 128.9, 128.4, 123.0, 120.3, 113.6, 113.3, 56.1, 56.0, 42.4, 33.3. Anal. calcd. for C_24_H_24_N_2_O_4_: C, 71.27; H, 5.98; N, 6.93. Found: C, 71.49; H, 6.19; N, 6.87.

*N-(4,5-Dimethoxy-2-(2-phenylacetyl)phenethyl)acetamide* (**3m**): known compound [44].

*N-(4,5-Dimethoxy-2-(2-phenylacetyl)phenethyl)benzamide* (**3n**): known compound [44].

### 3.2. Typical Procedure for the Preparation of Compounds ***4a-n***

To solution of the corresponding ketoamide **3** (1 mmol) in methanol (15 mL), NaBH_4_ (2 mmol, 0.1 g) was added portionwise. The solution was stirred 30 min at room temperature, than the solvent was removed under vacuum. Water (30 mL) was added to the residue and the solution was extracted with CH_2_Cl_2_ (3 × 20 mL), then the combined extracts were dried (Na_2_SO_4_). The products, after evaporation of the solvent, were obtained with 85–90% yields.

*N-(2-(1-Hydroxyethyl)-4,5-dimethoxyphenethyl)acetamide* (**4a**): ^1^H-NMR: 1.48 (d, *J* = 6.4, 3H), 1.85 (s, 3H), 2.72 (td, *J* = 7.1, 14.0, 1H), 2.86 (td, *J* = 6.9, 13.9, 1H), 3.13 (broad s, 1H, OH), 3.32 (td, *J* = 6.9, 13.3, 1H), 3.49 (td, *J* = 7.0, 13.4, 1H), 3.82 (s, 3H), 3.85 (s, 3H), 5.07 (q, *J* = 6.4, 1H), 6.28 (t, *J* = 4.6, 1H, NH), 6.60 (s, 1H), 7.00 (s, 1H); ^13^C-NMR: 170.5, 148.0, 147.8, 135.8, 127.9, 112.9, 108.9, 65.8, 55.9, 55.88, 41.0, 31.6, 24.4, 23.0. Anal. calcd. for C_14_H_21_NO_4_: C, 62.90; H, 7.92; N, 5.24. Found: C, 63.15; H, 8.06; N, 5.13.

*N-(2-(1-Hydroxyethyl)-4,5-dimethoxyphenethyl)benzamide* (**4b**): ^1^H-NMR: 1.84 (s, 3H), 2.07 (broad s, 1H, OH), 2.72 (td, *J* = 7.2, 14.1, 1H), 2.90 (td, *J* = 7.2, 14.1, 1H), 3.27–3.51 (m, 2H), 3.75 (s, 3H), 3.86 (s, 3H), 6.04 (d, *J* = 1.7, 1H), 6.18 (t, *J* = 4.9, 1H, NH), 6.67 (s, 1H), 6.83 (s, 1H), 7.25–7.35 (m, 5H); ^13^C-NMR: 170.6, 148.8, 147.5, 143.8, 134.2, 129.1, 128.3, 127.2, 126.5, 113.0, 111.4, 72.4, 55.9, 55.8, 40.9, 31.7, 22.9. Anal. calcd. for C_19_H_23_NO_4_: C, 69.28; H, 7.04; N, 4.25. Found: C, 69.43; H, 7.25; N, 4.13.

*N-(2-(1-Hydroxyethyl)-4,5-dimethoxyphenethyl)-2-phenylacetamide* (**4c**): ^1^H-NMR: 1.44 (d, *J* = 6.4, 3H), 2.76 (ddd, *J* = 7.1, 14.3, 32.3, 2H), 3.08 (broad s, 1H, OH), 3.43 (dtd, *J* = 7.4, 13.6, 13.4, 20.6, 2H), overpalled with 3.43 (s, 2H), 3.78 (s, 3H), 3.85 (s, 3H), 5.02 (q, *J* = 6.3, 6.4, 1H), 6.09 (t, *J* = 5.6, 1H, NH), 6.52 (s, 1H), 6.99 (s, 1H), 7.11–7.15 (m, 2H), 7.24–7.31 (m, 3H); ^13^C-NMR: 171.2, 147.8, 147.7, 135.9, 134.7, 129.2, 128.7, 127.5, 127.0, 112.8, 108.9, 65.6, 55.8, 43.4, 40.7, 31.4, 24.2. Anal. calcd. for C_20_H_25_NO_4_: C, 69.95; H, 7.34; N, 4.08. Found: C, 70.15; H, 7.53; N, 3.96.

*Ethyl2-(1-hydroxyethyl)-4,5-dimethoxyphenethylcarbamate* (**4d**): ^1^H-NMR: 1.19 (t, *J* = 7.1, 3H), 1.47 (d, *J* = 6.4, 3H), 2.62-2.90 (m, 2H), 3.36 (tt, *J* = 6.9, 13.6, 2H), 3.84 (s, 3H), 3.86 (s, 3H), 4.05 (q, *J* = 7.1, 2H), 4.92 (broad s, 1H, NH), 5.10 (q, *J* = 6.3, 1H), 6.60 (s, 1H), 7.03 (s, 1H); ^13^C-NMR: 156.8, 148.0, 148.0, 136.1, 127.4, 112.9, 108.8, 65.9, 60.8, 55.93, 55.91, 42.2, 32.4, 24.6, 14.6. Anal. calcd. for C_15_H_23_NO_5_: C, 60.59; H, 7.80; N, 4.71. Found: C, 60.73; H, 7.98; N, 4.56.

*N-(2-(1-Hydroxyethyl)-4,5-dimethoxyphenethyl)methanesulfonamide* (**4e**): ^1^H-NMR: 1.48 (d, *J* = 6.4, 3H), 1.91 (broad s, 1H, OH), 2.74 (s, 3H), 2.82 (td, *J* = 3.9, 6.7, 2H), 3.22–3.42 (m, 2H), 3.84 (s, 3H), 3.86 (s, 3H), 5.06 (q, *J* = 6.4, 1H), 5.31 (t, *J* = 6.5, 1H, NH), 6.65 (s, 1H), 6.99 (s, 1H); ^13^C-NMR: 148.3, 148.0, 135.7, 127.4, 112.9, 109.1, 66.0, 56.0, 55.9, 44.6, 39.9, 32.2, 24.4. Anal. calcd. for C_13_H_21_NO_5_S: C, 51.47; H, 6.98; N, 4.62; S, 10.57. Found: C, 51.68; H, 7.18; N, 4.44; S, 10.69.

*1-(2-(1-Hydroxyethyl)-4,5-dimethoxyphenethyl)-3-phenylurea* (**4f**): ^1^H-NMR: 1.43 (d, *J* = 6.4, 3H), 2.76 (q, *J* = 6.9, 1H), 2.87 (td, *J* = 7.1, 14.1, 1H), 3.31 (td, *J* = 7.0, 12.4, 1H), 3.58 (dq, *J* = 6.5, 13.3, 1H), 3.83 (s, 3H), 3.87 (s, 3H), 5.10 (dq, *J* = 3.5, 6.3, 1H), 5.80 (t, *J* = 5.7, 1H, NH), 6.66 (s, 1H), 6.93 (tt, *J* = 1.1, 7.5, 1H), 7.11 (s, 1H), 7.18–7.25 (m, 2H), 7.35 (dd, *J* = 1.1, 8.6, 2H); ^13^C-NMR: 155.7, 147.4, 147.3, 139.6, 136.8, 128.3, 127.6, 121.4, 118.2, 112.4, 108.6, 65.0, 55.5, 40.8, 31.8, 24.5. Anal. calcd. for C_19_H_24_N_2_O_4_: C, 66.26; H, 7.02; N, 8.13. Found: C, 66.47; H, 7.15; N, 8.01.

*N-(2-(Hydroxy(phenyl)methyl)-4,5-dimethoxyphenethyl)acetamide* (**4g**): ^1^H-NMR: 1.84 (s, 3H), 2.07 (broad s, 1H, OH), 2.72 (td, *J* = 7.2, 14.1, 1H), 2.90 (td, *J* = 6.8, 13.8, 1H), 3.27–3.51 (m, 2H), 3.75 (s, 3H), 3.86 (s, 3H), 6.04 (d, *J* = 1.7, 1H), 6.18 (t, *J* = 4.9, 1H, NH), 6.68 (s, 1H), 6.83 (s, 1H), 7.25–7.39 (m, 5H); ^13^C-NMR: 170.6, 148.3, 147.5, 143.7, 134.2, 129.1, 128.3, 127.2, 126.5, 113.0, 111.4, 72.4, 56.0, 55.8, 40.9, 31.7, 23.0. Anal. calcd. for C_19_H_23_NO_4_: C, 69.28; H, 7.04; N, 4.25. Found: C, 69.19; H, 7.24; N, 4.37.

*N-(2-(Hydroxy(phenyl)methyl)-4,5-dimethoxyphenethyl)benzamide* (**4h**): ^1^H-NMR: 1.81 (broad s, 1H, OH), 2.86 (td, *J* = 7.2, 14.3, 1H), 3.02 (td, *J* = 7.2, 14.0, 1H), 3.60 (dt, *J* = 7.2, 13.5, 2H), 3.75 (s, 3H), 3.80 (s, 3H), 6.0 (s,1H), 6.11 (broad s, 1H, NH), 6.71 (s, 1H), 6.82 (s, 1H), 7.50–7.25 (m, 8H), 7.70–7.67 (m, 2H); ^13^C-NMR: 167.8, 148.4, 147.6, 143.6, 134.4, 134.0, 131.4, 129.2, 128.5, 128.4, 127.3, 126.8, 126.5, 113.2, 111.6, 72.7, 55.9, 55.8, 41.3, 31.7. Anal. calcd. for C_24_H_25_NO_4_: C, 73.64; H, 6.44; N, 3.58. Found: C, 73.85; H, 6.23; N, 3.79.

*N-(2-(Hydroxy(phenyl)methyl)-4,5-dimethoxyphenethyl)-2-phenylacetamide* (**4i**): ^1^H-NMR: 1.90 (broad s, 1H, OH), 2.71–2.59 (m, 1H), 2.88 (ddd, *J* = 6.2, 7.4, 13.7, 1H), 3.54–3.25 (m, 2H), overlapped with 3.46 (s, 2H), 3.78 (s, 3H), 3.83 (s, 3H), 5.84 (t, *J* = 5.9, 1H, NH), 5.98 (d, *J* = 3.4, 1H), 6.59 (s, 1H), 6.82 (s, 1H), 7.17–7.14 (m, 2H), 7.37–7.25 (m, 8H); ^13^C-NMR: 171.4, 148.2, 147.6, 143.6, 134.8, 134.2, 129.4, 128.9, 128.7, 128.3, 127.2, 126.5, 113.1, 111.5, 72.4, 55.9, 55.8, 43.6, 40.7, 31.8. Anal. calcd. for C_25_H_27_NO_4_: C, 74.05; H, 6.71; N, 3.45. Found: C, 74.35; H, 6.97; N, 3.26.

*Ethyl 2-(hydroxy(phenyl)methyl)-4,5-dimethoxyphenethylcarbamate* (**4j)**: ^1^H-NMR: 1.18 (t, *J* = 7.1, 3H), 2.70 (td, *J* = 7.4, 14.0, 1H), 2.89 (td, *J* = 6.9, 13.4, 1H), 3.07 (broad s, 1H, OH), 3.29 (dd, *J* = 8.7, 13.8, 2H), 3.76 (s, 3H), 3.85 (s, 3H), 4.04 (q, *J* = 7.1, 2H), 4.83 (t, *J* = 6.6, 1H, NH), 6.03 (s, 1H), 6.65 (s, 1H), 6.87 (s, 1H), 7.21–7.37 (m, 5H); ^13^C-NMR: 156.8, 148.3, 147.7, 142.6, 134.1, 128.7, 128.4, 128.3, 127.3, 126.6, 113.1, 111.2, 72.4, 60.8, 55.92, 55.89, 42.0, 32.6, 14.6. Anal. calcd. for C_20_H_25_NO_5_: C, 66.83; H, 7.01; N, 3.90. Found: C, 67.01; H, 7.26; N, 3.85.

*N-(2-(Hydroxy(phenyl)methyl)-4,5-dimethoxyphenethyl)methanesulfonamide* (**4k**): ^1^H-NMR: 1.48 (d, *J* = 6.4, 3H), 1.80 (broad s, 1H, OH), 2.74 (s, 3H), 2.79 (dd, *J* = 6.9, 13.8, 1H), 2.91 (td, *J* = 7.0, 14.0, 1H), 3.26 (dd, *J* = 5.9, 12.7, 2H), 3.76 (s, 3H), 3.86 (s, 3H), 5.12 (t, *J* = 5.7, 1H, NH), 6.00 (d, *J* = 1.8, 1H), 6.69 (s, 1H), 6.83 (s, 1H), 7.23–7.33 (m, 5H); ^13^C-NMR: 148.6, 147.7, 143.4, 134.0, 128.4, 127.5, 126.5, 113.1, 111.5, 72.7, 56.0, 55.9, 44.4, 39.9, 32.4. Anal. calcd. for C_18_H_23_NO_5_S: C, 59.16; H, 6.34; N, 3.83; S, 8.77. Found: C, 59.38; H, 6.15; N, 3.93; S, 8.95.

*1-(2-(Hydroxy(phenyl)methyl)-4,5-dimethoxyphenethyl)-3-phenylurea* (**4l**): ^1^H-NMR: 2.0 (broad s, 1H, OH), 2.66 (td, *J* = 6.7, 13.7, 1H), 2.88 (td, *J* = 7.0, 14.0, 1H), 3.34 (dq, *J* = 6.1, 13.3, 2H), 3.68 (s, 3H), 3.78 (s, 3H), 4.26 (s, 1H, NH), 5.53 (t, *J* = 6.1, 1H, NH), 6.02 (s, 1H), 6.62 (s, 1H), 6.77 (s, 1H), 6.97–7.04 (m, 1H), 7.17–7.31 (m, 9H); ^13^C-NMR: 156.4, 148.3, 147.5, 143.6, 138.6, 134.3, 129.3, 129.0, 128.3, 127.2, 126.4, 123.3, 120.4, 113.0, 111.5, 72.3, 55.9, 55.8, 41.5, 32.4. Anal. calcd. for C_24_H_26_N_2_O_4_: C, 70.92; H, 6.45; N, 6.89. Found: C, 71.23; H, 6.27; N, 6.95.

*N-(2-(1-Hydroxy-2-phenylethyl)-4,5-dimethoxyphenethyl)acetamide* (**4m**): ^1^H-NMR: 1.86 (s, 3H), 1.98 (broad s, 1H, OH), 2.65 (dt, *J* = 7.0, 14.0, 1H), 2.78 (dt, *J* = 7.0, 13.9, 1H), 3.06 (dd, *J* = 3.8, 6.6, 2H), 3.25 (dt, *J* = 7.0, 12.9, 1H), 3.44 (qd, *J* = 6.8, 6.9, 13.4, 1H), 3.86 (s, 3H), 3.89 (s, 3H), 5.01 (t, *J* = 6.7, 1H), 5.93(t, *J* = 4.9, 1H, NH), 6.60 (s, 1H), 7.05 (s, 1H), 7.17–7.30 (m, 5H); ^13^C-NMR: 170.4, 148.2, 147.8, 138.0, 133.8, 129.5, 128.5, 128.2, 126.6, 112.6, 109.6, 71.6, 55.9, 45.2, 40.7, 31.4, 23.0. Anal. calcd. for C_20_H_25_NO_4_: C, 69.95; H, 7.34; N, 4.08. Found: C, 70.17; H, 7.56; N, 3.89.

*N-(2-(1-Hydroxy-2-phenylethyl)-4,5-dimethoxyphenethyl)benzamide* (**4n**): ^1^H-NMR: 1.87 (broad s, 1H, OH), 2.65 (td, *J* = 4.7, 16.6, 1H), 2.88 (ddd, *J* = 6.3, 7.4, 13.6, 1H), 3.54–3.24 (m, 2H), overlapped with 3.45-3.47 (m, 2H), 3.78 (s, 3H), 3.83 (s, 3H), 5.83 (t, *J* = 5.7, 1H, NH), 5.98 (s, 1H), 6.59 (s, 1H), 6.82 (s, 1H), 7.17–7.14 (m, 2H), 7.37–7.22 (m, 8H); ^13^C-NMR: 171.4, 148.2, 147.6, 143.7, 134.8, 134.2, 129.4, 128.9, 128.7, 128.3, 127.2, 126.5, 113.1, 111.5, 72.4, 55.9, 55.8, 43.6, 40.7, 31.8. Anal. calcd. for C_25_H_27_NO_4_: C, 74.05; H, 6.71; N, 3.45. Found: C, 73.81; H, 6.43; N, 3.49.

### 3.3. Typical Procedure for the Preparation of Compounds ***5***

In a 100-mL round-bottomed flask fitted with a glass-rod, stirrer, reflux condenser and inlet for argon are placed magnesium turnings (0.36 g, 15 mmol) in dry diethyl ether (30 mL). The apparatus is flushed with argon. A slow stream of argon is introduced. About one-fifth of a solution of the corresponding halide (15 mmol) in dry ether (20 mL) is added to the vigorously stirred mixture. Reaction commences within 2–8 minutes and the remainder of the halide solution is then added steadily over about 12 minutes to the mixture. Stirring is continued for an additional 60 minutes followed by adding of solution of corresponding ketoamide **3** (3 mmol) in CH_2_Cl_2 _(10 mL). The reaction mixture is poured into water (the end of the reaction is proved with thin-layer chromatography) and extracted with CH_2_Cl_2_ (4 × 20 mL). If emulsion was formed, the saturated solution of ammonium chloride is added. The combined extracts were dried (Na_2_SO_4_) and concentrated. The corresponding product were isolated after column chromatography on silicagel with n-hexane:diethyl ether as eluent with 50–65% yields.

*N-(2-(2-Hydroxypropan-2-yl)-4,5-dimethoxyphenethyl)acetamide* (**5a**): ^1^H-NMR: 1.67 (s, 6H), 1.89 (s, 3H), 2.61 (broad s, 1H, OH), 3.14 (t, *J* = 7.1, 2H), 3.49 (dd, *J* = 12.4, 6.8, 2H), 3.87 (s, 6H), 5.31 (s, 1H, NH), 6.73 (s, 1H), 6.89 (s, 1H); ^13^C-NMR: 170.4, 147.7, 146.5, 138.0, 129.8, 114.7, 109.8, 56.0, 55.85, 42.1, 32.6, 29.4, 23.1. Anal. calcd. for C_15_H_23_NO_4_: C, 64.03; H, 8.24; N, 4.98. Found: C, 64.25; H, 8.05; N, 4.77.

*N-(2-(2-Hydroxypropan-2-yl)-4,5-dimethoxyphenethyl)benzamide* (**5b**): ^1^H-NMR: 1.71 (s, 6H), 2.74 (s, 1H, OH), 3.24 (t, *J* = 6.8, 2H), 3.69 (dd, *J* = 11.7, 6.9, 2H), 3.81 (s, 3H), 3.85 (s, 3H), 6.74 (s, 1H, NH), 6.86 (s, 1H), 7.26 (s, 1H), 7.33–7.42 (m, 3H), 7.69 (d, *J* = 1.6, 1H), 7.72 (t, *J* = 1.4, 1H); ^13^C-NMR: 167.6, 147.8, 146.6, 137.7, 134.6, 131.1, 129.98, 128.3, 126.9, 114.7, 109.8, 56.1, 55.8, 42.6, 32.7, 32.2. Anal. calcd. for C_20_H_25_NO_4_: C, 69.95; H, 7.34; N, 4.08. Found: C, 69.64; H, 7.33; N, 4.17.

*N-(2-(2-Hydroxypropan-2-yl)-4,5-dimethoxyphenethyl)-2-phenylacetamide* (**5c**): ^1^H-NMR: 1.58 (s, 6H), 1.88 (s, 1H, OH), 3.08 (t, *J* = 6.9, 2H), 3.49-3.53 (m, 4H), 3.85 (s, 3H), 3.88 (s, 3H), 6.68 (s, 1H, NH), 6.81 (s, 1H), 7.14 (s, 1H), 7.15–7.18 (m, 2H), 7.29–7.33 (m, 3H); ^13^C-NMR: 171.3, 147.7, 146.4, 137.9, 135.1, 129.7, 128.8, 127.1, 114.5, 109.6, 56.1, 55.8, 43.7, 42.1, 32.3, 31.9. Anal. calcd. for C_21_H_27_NO_4_: C, 70.56; H, 7.61; N, 3.92. Found: C, 70.34; H, 7.56; N, 4.22.

*N-(2-(2-Hydroxy-1-phenylpropan-2-yl)-4,5-dimethoxyphenethyl)benzamide* (**5n**): ^1^H-NMR: 1.65 (s, 3H), 2.65 (s, 1H, OH), 3.03 (td, *J* = 5.5, 13.2, 1H), 3.12(d, *J* = 13.2, 1H), 3.21 (d, *J* = 13.2, 1H), 3.50 (ddd, *J* = 6.2, 8.6, 14.4, 1H), 3.70 (tt, *J* = 4.5, 8.9, 2H), 3.78 (s, 3H), 3.83 (s, 3H), 6.70 (s, 1H), 6.76 (s, 1H), 7.08 (dd, *J* = 1.6, 8.2, 2H), 7.24-7.28 (m, 3H), 7.33 (t, *J* = 7.7, 2H) 7.41 (t, *J* = 7.4, 1H), 7.63 (t, *J* = 4.7, 1H, NH), 7.71 (dd, *J* = 1.1, 8.4, 2H); ^13^C-NMR: 167.5, 147.9, 146.5, 136.7, 136.3, 134.6, 131.1, 130.8, 130.3, 128.3, 128.2, 128.19, 127.0, 126.9, 114.5, 110.4, 76.2, 56.1, 55.7, 50.5, 42.8, 31.9, 30.1. Anal. calcd. for C_26_H_29_NO_4_: C, 74.44; H, 6.97; N, 3.34. Found: C, 74.25; H, 6.77; N, 3.53.

*N-(2-(2-Hydroxybutan-2-yl)-4,5-dimethoxyphenethyl)benzamide* (**5p**): ^1^H-NMR: 0.92 (t, *J* = 7.4, 3H), 1.71 (s, 3H), 1.76 (ddd, *J* = 0.9, 2.0, 6.6, 2H), 2.90 (dt, *J* = 4.6, 7.0, 2H), 3.70 (ddd, *J* = 5.7, 7.1, 19.5, 2H), 3.85 (s, 3H), 3.90 (s, 3H), 6.22 (broad s, 1H, NH), 6.64 (s, 1H), 6.75 (s, 1H), 7.42–7.49 (m, 3H), 7.70–7.79 (m, 2H); ^13^C-NMR: 167.5, 147.6, 138.6, 136.3, 131.4, 130.1, 128.6, 128.4, 126.8, 124.4, 112.5, 110.8, 106.0, 63.9, 56.2, 55.8, 41.4, 32.3, 18.8, 12.4. Anal. calcd. for C_21_H_27_NO_4_: C, 70.56; H, 7.61; N, 3.92. Found: C, 70.26; H, 7.43; N, 4.13.

*N-(2-(1-Hydroxy-1-phenylethyl)-4,5-dimethoxyphenethyl)benzamide* (**5s**): ^1^H-NMR: 1.98 (s, 3H), 2.68 (t, *J* = 6.9, 2H), 2.79 (dd, *J* = 7.3, 13.8, 1H), 3.27 (dt, *J* = 6.2, 13.4, 1H), 3.84 (s, 3H), 3.92 (s, 3H), 5.80 (d, *J* = 1.3,1H, NH) 6.77 (s, 1H), 7.15 (s, 1H), 7.24–7.48 (m, 10H); ^13^C-NMR: 167.6, 149.3, 148.8, 147.4, 146.3, 134.6, 131.4, 126.5, 125.3, 115.5, 114.6, 113.8, 112.7, 110.9, 56.2, 55.9, 41.4, 40.7, 33.6. Anal. calcd. for C_25_H_27_NO_4_: C, 74.05; H, 6.71; N, 3.45. Found: C, 74.32; H, 6.97; N, 3.37.

*N-(2-(1-Hydroxy-1-phenylpropyl)-4,5-dimethoxyphenethyl)methanesulfonamide* (**5t**): ^1^H-NMR: 0.90 (t, *J* = 7.3, 3H), 2.30 (dq, *J* = 7.3, 13.8, 2H), 2.53 (broad s, 1H, OH), 2.67 (s, 3H), 2.73 (dd, *J* = 4.9, 9.0, 2H), 2.86 (td, *J* = 5.9, 17.8, 1H), 2.94–3.04 (m, 1H), 3.88 (s, 3H), 3.94 (s, 3H), 4.60 (t, *J* = 5.3, 1H, NH), 6.66 (s, 1H), 7.18 (s, 1H), 7.20–7.32 (m, 5H); ^13^C-NMR: 148.2, 147.2, 146.5, 137.1, 130.4, 127.9, 126.7, 125.9, 114.7, 111.1, 105.9, 78.6, 56.2, 55.9, 44.4, 39.7, 36.2, 32.7, 8.1. Anal. calcd. for C_20_H_27_NO_5_S: C, 61.05; H, 6.92; N, 3.56; S, 8.15. Found: C, 60.81; H, 6.64; N, 3.65; S, 7.83.

### 3.4. Cyclization of ***4*** and ***5*** to the Corresponding Isoquinolines ***6*** and ***7***

To solution of the corresponding compound **4** or **5** (1 mmol) in dichloromethane (15 mL) a catalytic amount of *p*-toluensulfonic acid (PTSA) was added. The solution was stirred 30 min at room temperature, then the solution was filtered on a short column with neutral Al_2_O_3_. The products **6** or **7**, after evaporation of the solvent, were obtained with 88–90% yields. When the substituent at the C-1 is ethyl or benzyl, the styrene products **8** were formed predominantly (~60%) than expected cyclic 1,1-disubtituted product **7 **(~30%). 

*1-(6,7-Dimethoxy-1-methyl-3,4-dihydroisoquinolin-2(1H)-yl)ethanone* (**6а**): known compound [49,50,51].

*(6,7-Dimethoxy-1-methyl-3,4-dihydroisoquinolin-2(1H)-yl)(phenyl)methanone* (**6b**): ^1^H-NMR (600 MHz): 1.60 (d, *J* = 6.7, 3H), 2.63 (d, *J* = 16.7, 1H), 2.94 (ddd, *J* = 5.3, 12.0, 17.3, 1H), 3.16 (td, *J* = 14.0, 30.6, 1H), 3.44 (dt, *J* = 3.2, 13.1, 1H), 3.88 (s, 6H), 5.72 (q, *J* = 5.7, 1H), 6.61 (s, 1H), 6.69 (s, 1H), 7.43–7.45 (m, 5H); ^13^C-NMR: 170.2, 147.9, 147.7, 136.7, 136.6, 129.5, 128.6, 126.6, 111.2, 109.8, 56.0, 55.9, 53.4, 40.9, 29.2, 21.5. Anal. calcd. for C_19_H_21_NO_3_: C, 73.29; H, 6.80; N, 4.50. Found: C, 72.95; H, 6.98; N, 4.45.

*1-(6,7-Dimethoxy-1-methyl-3,4-dihydroisoquinolin-2(1H)-yl)-2-phenylethanone* (**6c**): ^1^H-NMR (600 MHz): 1.40 (d, *J* = 6.8, 1H), 1.46 (d, *J* = 6.8, 3H), 2.52–2.60 (m, 2H), 3.40 (ddd, *J* = 5.0, 10.5, 13.5, 1H), 3.83 (d, *J* = 4.3, 2H), 3.85 (s, 3H), 3.87 (s, 3H), 5.63 (q, *J* = 6.8, 1H), 6.53 (s, 1H), 6.62 (s, 1H), 7.23–7.36 (m, 5H); ^13^C-NMR: 169.3, 147.9, 147.5, 135.2, 130.4, 128.9, 128.7, 126.8, 111.0, 109.8, 56.0, 55.9, 52.2, 41.8, 40.0, 28.8, 21.4. Anal. calcd. for C_20_H_23_NO_3_: C, 73.82; H, 7.12; N, 4.30. Found: C, 73.57; H, 7.31; N, 4.34.

*Ethyl 6,7-dimethoxy-1-methyl-3,4-dihydroisoquinoline-2(1H)-carboxylate* (**6d**): known compound [52,53,54,55].

*6,7-dimethoxy-1-methyl-2-(methylsulfonyl)-1,2,3,4-tetrahydroisoquinoline* (**6e**): known compound [56].

*6,7-dimethoxy-1-methyl-N-phenyl-3,4-dihydroisoquinoline-2(1H)-carboxamide* (**6f**): known compound [57].

*1-(6,7-dimethoxy-1-phenyl-3,4-dihydroisoquinolin-2(1H)-yl)ethanone* (**6g**): known compound [58,59,60].

*(6,7-dimethoxy-1-phenyl-3,4-dihydroisoquinolin-2(1H)-yl)(phenyl)methanone* (**6h**): known compound [58,59,60].

*1-(6,7-dimethoxy-1-phenyl-3,4-dihydroisoquinolin-2(1H)-yl)-2-phenylethanone* (**6i**): ^1^H-NMR (600 MHz): 2.61 (dd, *J* = 4.1, 7.7, 2H), 3.33 (td, *J* = 8.7, 14.3, 1H), 3.78 (s, 3H), overlapped with 3.74–3.79 (m, 1H), 3.83 (d, *J* = 2.0, 2H), 3.89 (s, 3H), 6.56 (s, 1H), 6.62 (s, 1H), 6.95 (s, 1H), 7.22–7.34 (m, 10H); ^13^C-NMR: 169.99, 147.6, 142.7, 142.5, 135.1, 128.8, 128.7, 128.2, 127.4, 126.7, 126.3, 111.4, 111.0, 92.2, 55.96, 55.88, 54.7, 41.4, 39.8, 28.4. Anal. calcd. for C_25_H_25_NO_3_: C, 77.49; H, 6.50; N, 3.61; Found: C, 77.68; H, 6.75; N, 3.56.

*Ethyl 6,7-dimethoxy-1-phenyl-3,4-dihydroisoquinoline-2(1H)-carboxylate* (**6j**): known compound [61,62,63].

*6,7-Dimethoxy-2-(methylsulfonyl)-1-phenyl-1,2,3,4-tetrahydroisoquinoline* (**6k**): ^1^H-NMR: 2.64 (s, 3H), 2.76 (ddd, *J* = 1.7, 4.5, 16.8, 1H), 3.09 (ddd, *J* = 6.3, 11.7, 16.3, 1H), 3.29 (ddd, *J* = 4.6, 11.8, 13.6, 1H), 3.77 (s, 3H), 3.82-3.91 (m, 1H), 3.92 (s, 3H), 6.02 (s, 1H), 6.48 (s, 1H), 6.71 (s, 1H), 7.27–7.36 (m, 5H); ^13^C-NMR: 148.5, 147.8, 140.9, 128.9, 128.5, 128.0, 125.8, 125.7, 111.5, 110.9, 58.7, 56.0, 55.9, 39.6, 38.5, 21.1. Anal. calcd. for C_18_H_21_NO_4_S: C, 62.23; H, 6.09; N, 4.03; S, 9.23. Found: C, 62.03; H, 6.27; N, 4.09; S, 9.15.

*6,7-Dimethoxy-N,1-diphenyl-3,4-dihydroisoquinoline-2(1H)-carboxamide* (**6l**): ^1^H-NMR (600 MHz): 1.56 (s, 1H), 2.71 (d, *J* = 16.4, 1H), 2.87 (dd, *J* = 7.3, 15.9, 1H), 3.51 (ddd, *J* = 2.9, 6.7, 11.7, 1H), 3.71 (s, 3H), 3.81 (s, 3H), 6.35 (s, 1H), 6.45 (broad s, 1H, NH), 6.55 (s, 1H), 6.61 (s, 1H), 6.95 (t, *J* = 7.2, 1H), 7.18–7.23 (m, 4H), 7.22–7.27 (m, 5H); ^13^C-NMR: 154.9, 148.1, 147.6, 142.7, 139.1, 128.9, 128.6, 127.9, 127.8, 127.6, 126.9, 123.1, 120.0, 111.2, 111.1, 57.4, 56.1, 56.0, 40.0, 28.1. Anal. calcd. for C_24_H_24_N_2_O_3_: C, 74.21; H, 6.23; N, 7.21. Found: C, 74.43; H, 6.45; N, 7.13.

*1-(1-Benzyl-6,7-dimethoxy-3,4-dihydroisoquinolin-2(1H)-yl)ethanone* (**6m**): ^1^H-NMR: 1.63 (s, 3H), 2.84 (ddd, *J* = 5.6, 10.1, 14.8, 2H), 3.09–3.17 (m, 2H), 3.50 (ddd, *J* = 3.4, 7.8, 10.8, 1H), 3.69 (td, *J* = 5.6, 12.9, 1H), 3.87 (s, 3H), 3.89 (s, 3H), 5.68 (dd, *J* = 5.3, 8.4, 1H), 6.14 (s, 1H), 6.48 (s, 1H), 7.17–7.27 (m, 5H); ^13^C-NMR: 169.5, 148.2, 147.3, 138.2, 129.6, 128.7, 128.2, 126.7, 125.5, 110.9, 110.1, 59.3, 56.0, 55.8, 43.2, 35.1, 28.6, 22.0. Anal. calcd. for C_20_H_23_NO_3_: C, 73.82; H, 7.12; N, 4.30. Found: C, 73.99; H, 7.05; N, 4.25.

*(1-Benzyl-6,7-dimethoxy-3,4-dihydroisoquinolin-2(1H)-yl)(phenyl)methanone* (**6n**): known compound [57].

*1-(6,7-Dimethoxy-1,1-dimethyl-3,4-dihydroisoquinolin-2(1H)-yl)ethanone* (**7a**): ^1^H-NMR: 1.82 (s, 6H), 2.21 (s, 3H), 2.79 (t, *J* = 5.5, 2H), 3.56 (dt, *J* = 5.5, 3.8, 2H), 3.87 (s, 3H), 3.89 (s, 3H), 6.57 (s, 1H), 6.77 (s, 1H); ^13^C-NMR: 170.3, 147.9, 147.1, 137.0, 126.3, 110.5, 109.6, 59.85, 56.1, 55.8, 44.1, 30.4, 27.99, 25.6. Anal. calcd. for C_15_H_21_NO_3_: C, 68.42; H, 8.04; N, 5.32. Found: C, 68.65; H, 8.18; N, 5.14. 

*(6,7-Dimethoxy-1,1-dimethyl-3,4-dihydroisoquinolin-2(1H)-yl)(phenyl)methanone* (**7b**): ^1^H-NMR: 1.96 (s, 6H), 2.80 (t, *J* = 5.5, 2H), 3.52 (dt, *J* = 5.5, 3.5, 2H), 3.89 (s, 3H), 3.92 (s, 3H), 6.59 (s, 1H), 6.84 (s, 1H), 7.42–7.46 (m, 3H), 7.48–7.52 (m, 2H); ^13^C-NMR: 172.6, 147.9, 147.2, 138.9, 136.8, 129.6, 128.5, 126.7, 126.1, 110.8, 109.7, 59.9, 56.1, 55.9, 45.3, 30.4, 27.7; Anal. calcd. for C_20_H_23_NO_3_: C, 73.82; H, 7.12; N, 4.30. Found: C, 73.87; H, 7.05; N, 4.57.

*1-(6,7-Dimethoxy-1,1-dimethyl-3,4-dihydroisoquinolin-2(1H)-yl)-2-phenylethanone* (**7c**): ^1^H-NMR: 1.84 (s, 6H), 2.49 (t, *J* = 5.3, 2H), 3.47 (dt, *J* = 4.8, 6.0, 2H), 3.82 (s, 3H), overlapped with 3.83 (s, 2Н), 3.86 (s, 3H), 6.47 (s, 1H), 6.75 (s, 1H), 7.23–7.27 (m, 2H), 7.30–7.33 (m, 3H); ^13^C-NMR: 170.9, 147.8, 147.1, 136.8, 135.6, 128.6, 128.4, 126.6, 126.3, 110.5, 109.6, 60.1, 56.1, 55.8, 44.8, 43.8, 30.1, 27.9; Anal. calcd. for C_21_H_25_NO_3_: C, 74.31; H, 7.42; N, 4.13. Found: C, 74.26; H, 7.38; N, 4.20.

*Ethyl 6,7-dimethoxy-1,1-dimethyl-3,4-dihydroisoquinoline-2(1H)-carboxylate* (**7d**): ^1^H-NMR: 1.32 (dd, *J* = 6.4, 13.5, 3H), 1.79 (s, 6H), 2.75 (t, *J* = 5.5, 2H), 3.73–3.77 (m, 2H), 3.88 (s, 3H), 3.89 (s, 3H), 4.19 (q, *J* = 7.12, 7.09, 2H), 6.57 (s, 1H), 6.78 (s, 1H); ^13^C-NMR: 181.6, 147.7, 147.1, 136.5, 127.0, 110.6, 109.8, 60.8, 58.6, 56.1, 55.8, 47.8, 30.2, 28.8, 14.6. Anal. calcd. for C_16_H_23_NO_4_: C, 65.51; H, 7.90; N, 4.77. Found: C, 65.84; H, 7.76; N, 4.80.

*6,7-Dimethoxy-1,1-dimethyl-2-(methylsulfonyl)-1,2,3,4-tetrahydroisoquinoline* (**7e**): ^1^H-NMR: 1.86 (s, 6H), 2.81 (dd, *J* = 5.0, 6.0, 2H), 2.98 (s, 3H), 3.67–3.62 (m, 2H), 3.86 (s, 3H), 3.87 (s, 3H), 6.54 (s, 1H), 6.71 (s, 1H); ^13^C-NMR: 147.8, 147.5, 135.6, 125.9, 110.9, 109.3, 61.4, 56.2, 55.8, 43.0, 42.6, 30.4, 30.0. Anal. calcd. for C_14_H_21_NO_4_S: C, 56.16; H, 7.07; N, 4.68; S, 10.71. Found: C, 56.36; H, 7.18; N, 4.63; S, 10.97.

*(1-Benzyl-6,7-dimethoxy-1-methyl-3,4-dihydroisoquinolin-2(1H)-yl)(phenyl)methanone* (**7n**): ^1^H-NMR: 2.09 (s, 3H), 2.27 (ddd, *J* = 2.7, 4.8, 15.2, 1H), 3.07 (ddd, *J* = 8.0, 12.6, 22.8, 1H), 3.21 (td, *J* = 4.1, 13.3, 1H), 3.88 (s, 3H), 3.97 (s, 3H), 4.56 (d, *J* = 13.3, 1H), 6.42 (s, 1H), 6.68 (dd, *J* = 1.5, 8.6, 2H), 7.00 (s, 1H), 7.05 (t, *J* = 7.5, 2H), 7.11–7.13 (m, 1H), 7.24 (d, *J* = 5.6, 2H), 7.35–7.38 (m, 3H); ^13^C-NMR: 172.4, 147.9, 147.3, 139.1, 137.8, 133.7, 130.1, 129.3, 129.0, 128.5, 127.5, 127.4, 126.4, 126.2, 126.15, 110.2, 109.8, 64.2, 56.3, 56.2, 45.9, 45.3, 29.7, 26.4. Anal. calcd. for C_26_H_27_NO_3_: C, 77.78; H, 6.78; N, 3.49. Found: C, 77.53; H, 6.87; N, 3.52.

*1-(1-Ethyl-6,7-dimethoxy-1-methyl-3,4-dihydroisoquinolin-2(1H)-yl)ethanone* (**7o**): ^1^H-NMR: 0.55 (t, *J* = 7.4, 3H), 1.65 (dt, *J* = 7.3, 14.6, 1H), 1.78 (s, 3H), 2.24–2.26 (m, 1H), 2.70 (ddd, *J* = 3.4, 5.3, 15.6, 1H), 2.86 (ddd, *J* = 4.9, 10.1, 13.0, 1H), 3.29 (dd, *J* = 7.4, 14.1, 1H), 3.42 (ddd, *J* = 3.3, 9.4, 13.0, 1H), 3.89 (s, 6H), 6.58 (s, 1H), 6.76 (s, 1H); ^13^C-NMR: 185.6, 148.0, 147.0, 134.6, 128.1, 110.2, 109.2, 63.9, 56.1, 55.8, 44.7, 33.4, 26.7, 25.5, 8.5. Anal. calcd. for C_16_H_23_NO_3_: C, 69.29; H, 8.36; N, 5.05. Found: C, 69.13; H, 8.48; N, 5.12.

*(1-Ethyl-6,7-dimethoxy-1-methyl-3,4-dihydroisoquinolin-2(1H)-yl)(phenyl)methanone* (**7p**): ^1^H-NMR: 0.66 (t, *J* = 7.3, 3H), 1.77 (dd, *J* = 7.2, 14.1, 1H), 1.93 (s, 3H), 2.60 (ddd, *J* = 3.1, 4.0, 15.3, 1H), 2.94 (ddd, *J* = 3.6, 10.5, 14.5, 1H), 3.31 (ddd, *J* = 2.8, 10.5, 13.3, 1H), 3.43 (dd, *J* = 7.2, 14.1, 1H), 3.77 (td, *J* = 4.0, 13.3, 1H), 3.90 (s, 3H), 3.93 (s, 3H), 6.59 (s, 1H), 6.81 (s, 1H), 7.43–7.51 (m, 5H); ^13^C-NMR: 172.0, 148.0, 147.2, 139.0, 129.4, 128.5, 128.0, 126.6, 110.5, 109.3, 63.8, 56.1, 55.8, 45.8, 33.0, 30.3, 26.4, 8.5. Anal. calcd. for C_21_H_25_NO_3_: C, 74.31; H, 7.42; N, 4.13. Found: C, 74.11; H, 7.64; N, 4.03.

*1-Ethyl-6,7-dimethoxy-1-methyl-2-(methylsulfonyl)-1,2,3,4-tetrahydroisoquinoline* (**7r**): ^1^H-NMR: 0.72 (t, *J* = 7.3, 3H), 1.77 (dd, *J* = 8.1, 15.4, 1H), 1.84 (s, 3H), 2.74–2.80 (m, 2H), 2.83-2.89 (m, 1H), 2.99 (s, 3H), 3.42 (ddd, *J* = 3.4, 8.8, 12.3, 1H), 3.72 (ddd, *J* = 4.1, 5.7, 12.4, 1H), 3.88 (s, 6H), 6.56 (s, 1H), 6.69 (s, 1H); ^13^C-NMR: 148.0, 147.4, 133.4, 127.6, 110.6, 108.9, 65.5, 56.2, 55.8, 43.6, 41.2, 36.2, 30.2, 28.2, 8.6. Anal. calcd. for C_15_H_23_NO_4_S: C, 57.48; H, 7.40; N, 4.47; S, 10.23. Found: C, 57.24; H, 7.62; N, 4.54; S, 10.35.

*(6,7-Dimethoxy-1-methyl-1-phenyl-3,4-dihydroisoquinolin-2(1H)-yl)(phenyl)methanone* (**7s**): ^1^H-NMR: 2.26 (s, 3H), 2.85 (ddd, *J* = 3.1, 5.4, 15.4, 1H), 3.21 (ddd, *J* = 4.1, 9.4, 14.7, 1H), 3.59–3.69 (m, 1H), overlapped with 3.64 (s, 3H), 3.88 (s, 3H), 3.97 (ddd, *J* = 4.3, 5.2, 13.3, 1H), 6.28 (s, 1H), 6.61 (s, 1H), 7.39-7.45 (m, 5H), 7.47-7.51 (m, 5H). Anal. calcd. for C_25_H_25_NO_3_: C, 77.49; H, 6.50; N, 3.61. Found: C, 77.14; H, 6.73; N, 3.65.

*1-Ethyl-6,7-dimethoxy-2-(methylsulfonyl)-1-phenyl-1,2,3,4-tetrahydroisoquinoline* (**7t**): ^1^H-NMR: 0.80 (t, *J* = 7.2, 3H), 1.90 (s, 3H), 1.98 (dd, *J* = 6.6, 13.8, 1H), 2.82 (td, *J* = 2.7, 15.4, 1H), 3.14 (ddd, *J* = 4.1, 11.6, 15.6, 1H), 3.30 (dt, *J* = 2.4, 11.1, 11.3, 1H), 3.59 (s, 3H), 3.64 (dd, *J* = 7.3, 13.8, 1H), 3.91 (s, 3H), 4.05 (td, *J* = 3.8, 7.1, 1H), 6.09 (s, 1H), 6.61 (s, 1H), 7.29–7.40 (m, 3H), 7.48–7.53 (m, 2H); ^13^C-NMR: 148.0, 147.5, 144.0, 133.3, 128.9, 128.2, 128.1, 127.8, 111.2, 109.5, 69.1, 56.0, 55.8, 43.2, 37.1, 34.5, 30.0, 6.3. Anal. calcd. for C_20_H_25_NO_4_S: C, 63.97; H, 6.71; N, 3.73; S, 8.54. Found: C, 64.41; H, 6.88; N, 3.76; S, 8.14.

*N-(2-(but-2-en-2-yl)-4,5-dimethoxyphenethyl)benzamide* (**8g**): ^1^H-NMR: 1.76 (ddd, *J* = 0.9, 2.0, 6.6, 1H), 1.96 (td, *J* = 1.2, 2.2, 1H), 2.90 (dt, *J* = 4.5, 7.01, 2H), 3.29 (tdd, *J* = 6.2, 12.7, 19.5, 2H), 3.85 (s, 3H), 3.89 (s, 3H), 5.41 (dq, *J* = 1.45, 6.7, 1H), 6.22 (broad s, 1H, NH), 6.40 (s, 1H), 6.75 (s, 1H), 7.42–7.49 (m, 3H), 7.71–7.75 (m, 2H); ^13^C-NMR: 167.5, 147.9, 147.2, 147.1, 138.6, 136.3, 131.4, 128.6, 128.5, 127.0, 124.4, 112.5, 112.4, 56.2, 55.9, 41.4, 32.4, 18.8, 13.9. Anal. calcd. for C_21_H_25_NO_3_: C, 74.31; H, 7.42; N, 4.13. Found: C, 74.12; H, 7.54; N, 4.15.

*N-(4,5-dimethoxy-2-(1-phenylprop-1-enyl)phenethyl)benzamide* (**8n**): ^1^H-NMR: 2.22 (d, *J* = 1.1, 3H), 2.95 (t, *J* = 7.1, 2H), 3.70 (dd, *J* = 6.9, 13.1, 2H), 3.84 (s, 3H), 3.89 (s, 3H), 6.23 (t, *J* = 4.7, 1H, NH), 6.39 (s, 1H), 6.73 (s, 1H), 6.78 (s, 1H), 7.34–7.39 (m, 7H, Ar), 7.44–7.47 (m, 1H, Ar), 7.68 (dd, *J* = 1.2, 8.4, 2H); ^13^C-NMR: 167.4, 147.9, 147.3, 138.4, 137.6, 131.4, 129.9, 128.9, 128.5, 128.3, 128.2, 127.5, 126.8, 126.7, 126. 66, 112.5, 111.8, 56.0, 55.8, 41.4, 32.5, 21.1. Anal. calcd. for C_26_H_27_NO_3_: C, 77.78; H, 6.78; N, 3.49. Found: C, 77.85; H, 6.89; N, 3.55.

*N-(4,5-dimethoxy-2-(1-phenylvinyl)phenethyl)benzamide* (**8s**): ^1^H-NMR: 2.69 (t, *J* = 7.2, 2H), 3.52 (dd, *J* = 7.0, 12.9, 2H), 3.88 (s, 3H), 3.89 (s, 3H), 5.25 (d, *J* = 1.3, 1H), 5.81 (d, *J* = 1.4, 1H), 6.00 (t, *J* = 6.1, 1H, NH), 6.80 (s, 1H), 6.81 (s, 1H), 7.28–7.31 (m, 4H), 7.38–7.50 (m, 4H), 7.65–7.69 (m, 2H); ^13^C-NMR: 167.3, 148.8, 148.5, 147.3, 140.7, 134.6, 133.8, 131.3, 129.0, 128.5, 127.9, 126.7, 126.5, 115.5, 114.6, 113.8, 112.7, 56.0, 55.9, 40.8, 32.7. Anal. calcd. for C_25_H_25_NO_3_: C, 77.49; H, 6.50; N, 3.61. Found: C, 77.64; H, 6.65; N, 3.55.

*N-(4,5-dimethoxy-2-(1-phenylprop-1-enyl)phenethyl)methanesulfonamide* (**8t**): ^1^H-NMR: 0.81 (t, *J* = 7.3, 3H), 2.09 (ddd, *J* = 6.2, 8.6, 12.6, 2H), 2.97 (s, 3H), 3.23 (td, *J* = 1.7, 7.1, 2H), 3.46–3.50 (m, 1H), 3.86 (s, 3H), 3.88 (s, 3H), 6.43 (s, 1H), 6.71 (s, 1H), 6.82–6.86 (m, 2H), 7.05-7.13 (m, 3H); ^13^C-NMR: 147.9, 147.3, 139.1, 137.8, 133.7, 130.1, 129.3, 129.0, 128.5, 127.4, 126.4, 126.2, 110.2, 109.8, 56.2, 55.9, 45.9, 45.3, 29.7, 26.4. Anal. calcd. for C_20_H_25_NO_4_S: C, 63.97; H, 6.71; N, 3.73; S, 8.54. Found: C, 63.76; H, 6.81; N, 3.77; S, 8.24.

### 3.5. Estimation of Contractile Activity of Some of the Newly Synthesized Compounds

#### 3.5.1. Collection and Preparation of Tissue Samples

Guinea-pigs (350 ± 50g) were used. Whole mount muscle preparations without mucosa were obtained from gastric corpus. The tissue was pinned flat in a dissecting dish containing preparation solution containing (mmol/L) Na^+^ - 143; K^+^ - 5.84; Ca^2+^ - 3.7 and preparations were cut longitudinal muscle fibres with a final size of (13.0 ± 1.5−1.0 ± 1.5) mm. Tissue samples were immediately rinsed with cooled (4 °C) preparation solution. The muscle preparations were suspended in fort individual organ baths containing 15 mL modified Krebs’ solution (KS) containing (mmol/L) Na^+^ - 143, K^+^ - 5.84, Ca^2+^ - 2.5, Mg^2+^ - 1.19, Cl^−^ - 133, HCO_3_^−^ - 16.7, H_2_PO_4_^−^ - 1.2 and 11.5 glucose (35.5 ± 0.25 °C) each and constantly oxygenated with 95% O_2_ and 5% CO_2_. The preparations were connected to an isometric force transducer (TRI 201, LSi LETICA; Pnlab s.l., Barcelona, Spain). Preparations were allocated to the organ baths in random manner and were allowed an equilibration period of 1 h. Muscle tension was preset to 7 mN g in one step during the equilibration time. The mechanical activity was amplified with 4-channels tensiometrical interface system for registration and investigation of spontaneous muscle contractility of the muscle strips.

#### 3.5.2. Estimation of SM Contractile Activity after Applications of Newly Synthesized Compounds

Preparations from 3 to 5 guinea-pigs were used for each experiment of studying of effect of application of isoquinoline derivatives. Basal tone (BT), frequency, mean amplitude (Amean) and area under the curve (AUC) were analysed after the equilibration time for a 5-min period. These values were defined as predrug (baseline period) and were used for further comparative analysis.

Again, at the end of each trial, the organ baths were flushed and 1 × 10^−5^ M acetylcholine was added to test the ability of the preparations to exert a contractile response after activation of cholinergic receptors. For each trial, four preparations from the same animal were used and compounds were assigned to the organ baths in random order.

The signal digitalisations ware achieved by 13 bit analogue to digital converter based on microcontroller. A logical level synchronization was developed by special controller for parallel PC port communication. The calibration curve was used to define the range of application of registration system 0–20 mN. 

Special Visual-Basic program was assembled to visualization of spontaneous smooth muscle activity with options to dynamically alteration of amplification, offset value and printing ability of smooth-muscle parameter. It is possible to build in the first deviation of the signals and saving of incoming data in 4 dimensional matrixes with appropriated format to statistical analysis.

#### 3.5.3. Parameters, Data Analysis and Statistics

The following parameters were analysed to describe contractility parameters for each application: BT, Amean, AUC and frequency. Basal tone of the muscle has been measured because an increase in this parameter can be independent of Amean of contractions or frequency of contractions. In addition to an increase or decrease in BT, changes in frequency of contractions or Amean indicate changes in contractility due to the drugs used. The variables were calculated by using the software ChartTM included in the PowerLab from ADInstruments Ltd, Australia. All results were expressed as percentage of the corresponding predrug measurement.

Statistical analysis was performed using Statistica 4.5 (StaSoft, Inc. Microsoft), SPSS Inc., Chicago, IL, USA, Excel VB for applications end PraphPad. Data were subjected to descriptive and comparative analyses. 

Data of are presented as mean and standard error (SEM), and 25 and 75% percentiles. Wilcoxon signed rank test was used to compare predrug between solvent and drug. In case of no significant difference in predrug between specimens used for solvent and drug, further calculations were performed. Differences within the results were analysed by the Friedman test. If Friedman analysis revealed significant differences for compound and the corresponding control, Wilcoxon signed rank test was used.

## 4. Conclusions

In conclusion, new isoquinoline derivatives were synthesized, as type of compounds found in nature and among bioactive compounds of interest. We have developed a convenient method for their synthesis by interaction of ketoamides with organomagnesium compounds, followed by cyclization in acidic medium with a catalytic amount of PTSA. A variety of substituents at the C-1 in the isoquinoline skeleton can be readily introduced. The estimation of contractile activity against smooth muscle preparations showed that some of the obtained compounds, especially 1,1-dialkyl 1,2,3,4-tetrahydroisoquinolines with sulfonamide substitutent possess the most pronounced effect.
